# Zwiesel bat banyangvirus, a potentially zoonotic Huaiyangshan banyangvirus (Formerly known as SFTS)–like banyangvirus in Northern bats from Germany

**DOI:** 10.1038/s41598-020-58466-w

**Published:** 2020-01-28

**Authors:** Claudia Kohl, Annika Brinkmann, Aleksandar Radonić, Piotr Wojtek Dabrowski, Andreas Nitsche, Kristin Mühldorfer, Gudrun Wibbelt, Andreas Kurth

**Affiliations:** 1 Robert Koch Institute, Centre for Biological Threats and Special Pathogens, Berlin, Germany; 2 Robert Koch Institute, Methodology and Research Infrastructure, Berlin, Germany; 3 Leibniz Institute for Zoo and Wildlife Research, Berlin, Germany

**Keywords:** Viral genetics, Pathogens, Virology

## Abstract

Bats are reservoir hosts for several emerging and re-emerging viral pathogens causing morbidity and mortality in wildlife, animal stocks and humans. Various viruses within the family *Phenuiviridae* have been detected in bats, including the highly pathogenic Rift Valley fever virus and Malsoor virus, a novel Banyangvirus with close genetic relation to Huaiyangshan banyangvirus (BHAV)(former known as Severe fever with thrombocytopenia syndrome virus, SFTSV) and Heartland virus (HRTV), both of which have caused severe disease with fatal casualties in humans. In this study we present the whole genome of a novel Banyangvirus, named Zwiesel bat banyangvirus, revealed through deep sequencing of the *Eptesicus nilssonii* bat virome. The detection of the novel bat banyangvirus, which is in close phylogenetic relationship with the pathogenic HRTV and BHAV, underlines the possible impact of emerging phenuiviruses on public health.

## Introduction

The Banyangvirus genus, currently classified as part of the *Phenuviridae* family in the order *Bunyavirales*, is characterized by a tri-segmented, negative- or ambisense ssRNA genome of 11–19 kb length in total. Four structural proteins are encoded on the genome: the L protein (RNA-dependent RNA polymerase) on segment L, two glycoproteins Gn and Gc on segment M, the nucleocapsid protein on segment N and the nonstructural protein NSs on segment S. Banyangviruses can infect vertebrates and invertebrates and have caused febrile infections, encephalitis and severe fevers with fatal outcome in humans, therefore being increasingly reported as emerging pathogens of public health importance^1–3^. Recently, two novel tick-borne phenuiviruses (Huaiyangshan banyangvirus [BHAV] and Heartland virus [HRTV]) were detected and characterized; they form, together with Guertu banyangvirus a new genus within the *Phenuiviridae* genus: the Banyangviruses clade^1,2^. BHAV was initially reported in 2011 in the Henan and Hubei Provinces, China. Patients showed fever or hemorrhagic fever, thrombocytopenia, leukocytopenia and multi-organ dysfunction with an initial case fatality rate of 30%^1,4^. By then, the etiological virus was isolated from patients’ blood and *Haemaphysalis longicornis* and *Rhipicephalus microplus* ticks throughout China, South Korea and Japan^5,6^. Similar symptoms were recognized in two men from Missouri, USA. The respective virus, named Heartland virus (HRTV), was isolated in 2012 from patients’ blood and *Amblyomma americanum* ticks collected in the field^2,7^. A third novel banyangvirus with close relation to BHAV and HRTV was detected in Tasmania State, Australia. The virus, named Hunter Island Group virus (HIGV), was isolated from *Ixodes eudyptidis* ticks which were blood-feeding on a shy albatross (*Thalassarche cauta*) colony with signs of unknown disease outbreak^8^. Despite the identification of ticks as vectors for BHAV, HRTV and HIGV, the amplifying or reservoir hosts of the viral pathogens remain unknown.

Bats are the primary reservoir host for several zoonotic viruses with a high impact on public health, including numerous filoviruses, paramyxoviruses, coronaviruses and bunyaviruses^9^. So far, two members of the family *Phenuiviridae* are associated with bats. The highly pathogenic Rift Valley fever virus was detected in *Micropteropus pusillus* and *Hipposideros caffer* bats in Guinea^10^. Malsoor virus, a fourth novel banyangvirus within the genus Banyangvirus, was recently isolated from a *Rousettus leschenaultii* bat in India^11^.

Bats may be the source of many more yet unknown viruses which can cause morbidity and mortality in wildlife, animal stocks and humans^12^. Novel sequencing technologies allow massive parallel sequencing of all organisms in one sample, also allowing the detection of viruses with low sequence similarity to already known viruses^13,14^.

In this study, organs of microchiropteran bats, which were moribund or found dead, with potentially virus-related histopathological changes were used for deep sequencing of the bat virome. A novel banyangvirus, named Zwiesel bat banyangvirus (ZbbV) after the town of bat origin in Germany, was detected in the virome of several Northern bats *(Eptesicus nilssonii)*. Phylogenetic analysis of the ZbbV polymerase protein showed that the closest relationship was to Malsoor virus, HRTV and BHAV.

## Results

The combination of sequences obtained from virome data and gap-filling PCR revealed the partial genome sequence of the novel ZbbV with 6,277, 3,434 and 1,793 nt for the L, M and S segment, respectively. The L segment open reading frame encodes for the RNA polymerase, the M segment for the two glycoproteins. Two open reading frames in ambisense orientation were determined on the S segment, which code the N protein (sense) and the nonstructural NSs protein (antisense).

Phylogenetic tree reconstruction of the ZbbV L, M and S segment revealed a monophyletic relationship with Malsoor virus, HRTV and BHAV within the genus banyangvirus. No signs of genetic reassortment were observed. Comparison of the L segment showed 84% aa (74% nt), 71% aa (67% nt) and 69% aa (66% nt) identity to Malsoor virus, HRTV and BHAV, respectively. Comparison of the M segment showed 70% aa (68% nt), 57% aa (58% nt) and 56% aa (59% nt) identity to Malsoor virus, HRTV and BHAV, respectively. Comparison of the S segment showed 38% aa (69% nt), 10% aa (48% nt) and 16% aa (49% nt) identity to Malsoor virus, HRTV and BHAV, respectively.

Using specifically designed primers for the ZbbV L gene and subsequent sequencing of the PCR products, ZbbV was detected and quantified by qPCR in liver, lung, brain, spleen and intestine of 5 out of 12 *Eptesicus nilssonii* bats used in this study (Table 1). qPCR screening of pooled organs from the remaining 363 bats, including 3 additional *Eptesicus nilssonii* bats, did not reveal any additional ZbbV sequences.

**Table 1:** Overview on bats used in the bat virome study, positive for Zwiesel bat banyangvirus.

Bat No.	Source	Organs used for metagenomics analysis (#)	Positive Organs (CT value)
E 143/09	94227 Zwiesel, Bavaria	Brain, Heart, Intestine, Lung, Spleen	Brain (35,34), Intestine, Spleen (39,21)
E 138/09	94258 Frauenau, Bavaria	Lung, Liver, Spleen, Intestine	Intestine (35,59), Liver (35,28), Spleen (35,89)
E 108/09	82319 Percha-Starnberg, Bavaria	Liver, Lung, Spleen	Liver (36,77), Lung (36,49), Spleen (35,28)
E 194/07	94267 Prackenbach, Bavaria	Liver, Lung, Spleen	Lung (34,93)
E 202/07	Bavaria	Liver, Lung, Spleen	Liver (38,91), Lung (36,49), Spleen (18,69)
E 145/09	94244 Geiersthal, Bavaria	Lung	
E 193/07	94258 Frauenau, Bavaria	Intestine, Lung, Spleen	
E 139/09	94155 Otterskirchen, Bavaria	Lung, Spleen	
E 196/07	Bavaria	Lung, Spleen	
E 141/09	94469 Deggendorf, Bavaria	Intestine, Lung	
E 147/09	94518 Spiegelau/Palmberg, Bavaria	Lung	
E 148/09	94469 Deggendorf, Bavaria	Spleen	
E 207/07	Bavaria		
E 6/09	92421 Maxhütte-Haidorf, Bavaria		
E 144/09	95028/30/32 Hof, Bavaria		

(#)Potentially virus-related histopathological changes.

The sequences of the L, M and S segments of the novel virus were deposited in GenBank with the accession numbers MN823639, MN823640, and MN823641, respectively. The novel virus was named Zwiesel bat banyangvirus (ZbbV), after the town of Zwiesel in Bavaria, where one of the *Eptesicus nilssonii* bats was found positive.

To obtain the virus isolate of the novel banyangvirus, Vero and Paki cell lines were inoculated with pooled organs of all 12 *Eptesicus nilssonii* bats. After 3 passages, no cytopathic effect was observed and cell culture supernatants were tested negative for ZbbV.

## Discussion

In this study we identified a novel banyangvirus, named Zwiesel bat banyangvirus, in the virome of *Eptesicus nilssonii* bats. Genetic characterization showed that the virus is related to the tick-borne zoonotic phenuiviruses BHAV and HRTV, responsible for severe disease and death in humans, as well as to Malsoor virus which was recently isolated from a *Rousettus* bat in India.

Since similar viruses have been detected in far apart countries such as North America, India, China, Japan, South Korea and Australia and in ticks of different genera, numerous zoonotic phenuiviruses may be widely distributed in different parts of the world. So far, ZbbV is the first banyangvirus detected in bats of northern Europe. Grouping of ZbbV within the tick-transmitted genus Banyangvirus suggests ticks to be the vector of the novel virus. Additionally, we have not found any evidence that ZbbV does encode for a NSm protein on segment M, this is corresponding with all other members of the genus Banyangvirus. As shown before, NSm is important for infectivity in *Aedes aegypti* mosquitoes^15^. However, although *Haemaphysalis longicornis* ticks are supposed to be the vector of BHAV, most infected patients did not report tick bites prior to disease onset^1^. Moreover, investigation of a cluster of BHAV cases suggests evidence of person-to-person transmission^15^, making the further study of BHAV- and BHAV-like viruses imperative regarding human public health.

It is tempting to suggest that viruses discovered in insectivorous bats are only remnants of the bats’ diet. Although we found sequences of ZbbV in the intestines of the bats, we also detected the virus in bat liver, lung, brain and spleen using specific PCR assays.

Considering the close relationship of ZbbV to BHAV and HRTV as well as the broad distribution of zoonotic phenuiviruses and *Eptesicus nilssonii* bats, which are the most abundant bats in northern Eurasia, the potential zoonotic impact of emerging banyangviruses on public health should be further investigated.

## Methods

### Study

In compliance with the species protection through the European Commission (https://ec.europa.eu/environment/nature/legislation/habitatsdirective/) and the Agreement on the Conservation of Populations of European Bats (www.eurobats.org), investigative research on bats was permitted by local government bodies. We herewith confirm, that all permits to investigate carcasses of deceased bats and all experimental protocols on the dead bats were approved by the respective local governmental authorities (district government of Upper Bavaria, Munich [No. 55.1-8642.1-4-2006]; district government of Bavarian Swabia, Augsburg [No. 51-8645.11/489]; Lower Saxony water management, coastal defence and nature conservation, Hannover [No. 897.21-20]; senate department for urban development and the environment, Berlin [No. I E 222 - 10.04.2004]). We herewith further confirm that all experiments were performed in accordance with relevant guidelines and regulations. All bats in a broad study on diseases of native bats in Germany (n = 375) were found dead or moribund with subsequent euthanization and were kindly provided by bat researchers and bat rehabilitation centers^16^. 12 *Eptesicus nilssonii* bats collected throughout urban and suburban areas of Bavaria, showed potentially virus-related histopathological changes and have been further examined in this study.

### Methods

**Figure 1:**
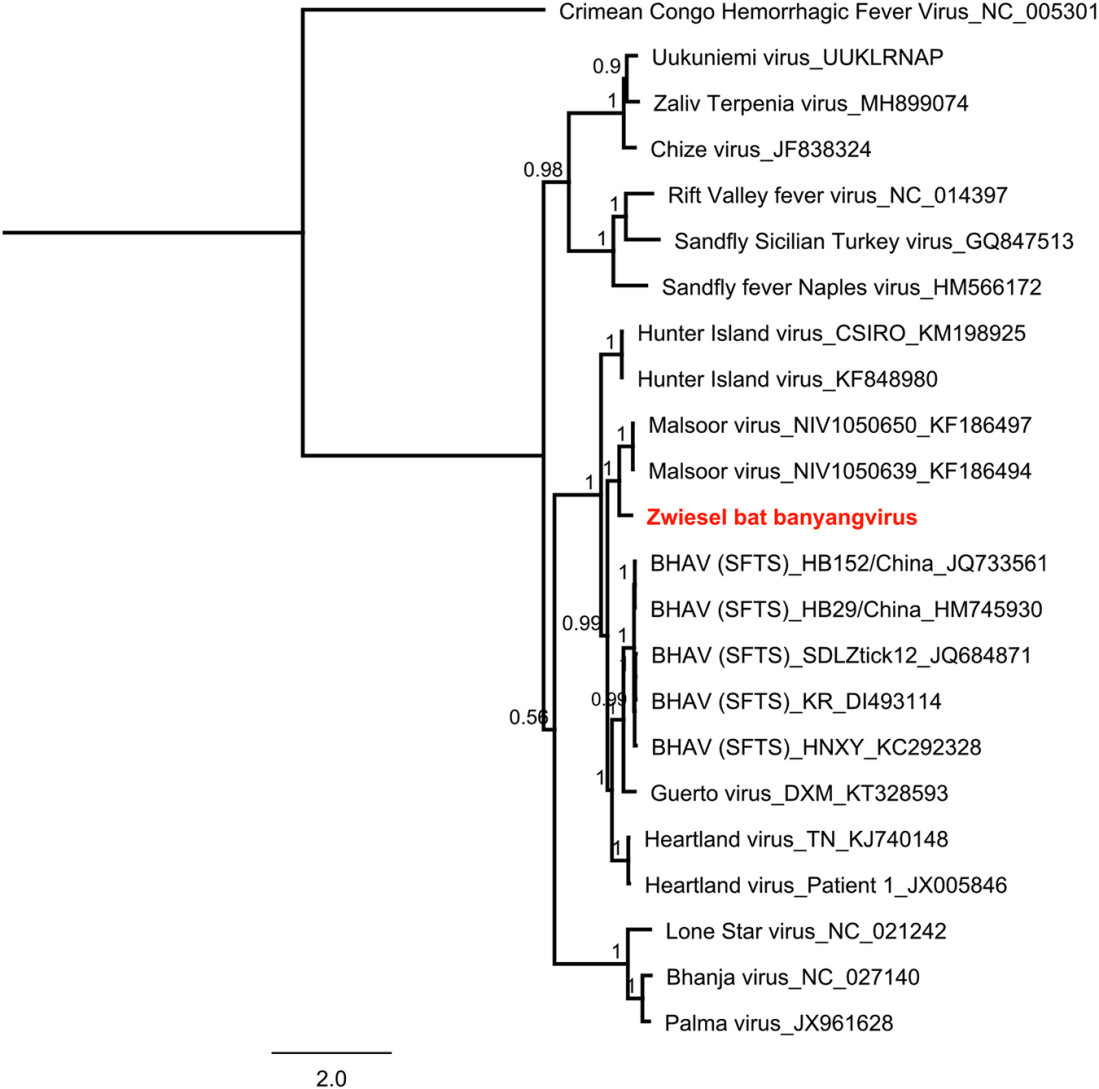
Phylogenetic analysis of the nucleotide sequence (6,022 nt) of the Zwiesel bat banyangvirus L segment). Alignments were built with ClustalW before model prediction with JModel-test. Phylogenetic tree reconstruction was calculated using MrBayes with 500,000 replicates, model GTR, sampling frequency 200, burn in 10% (50,000). Posterior probabilities are depicted at the branches.

**Figure 2:**
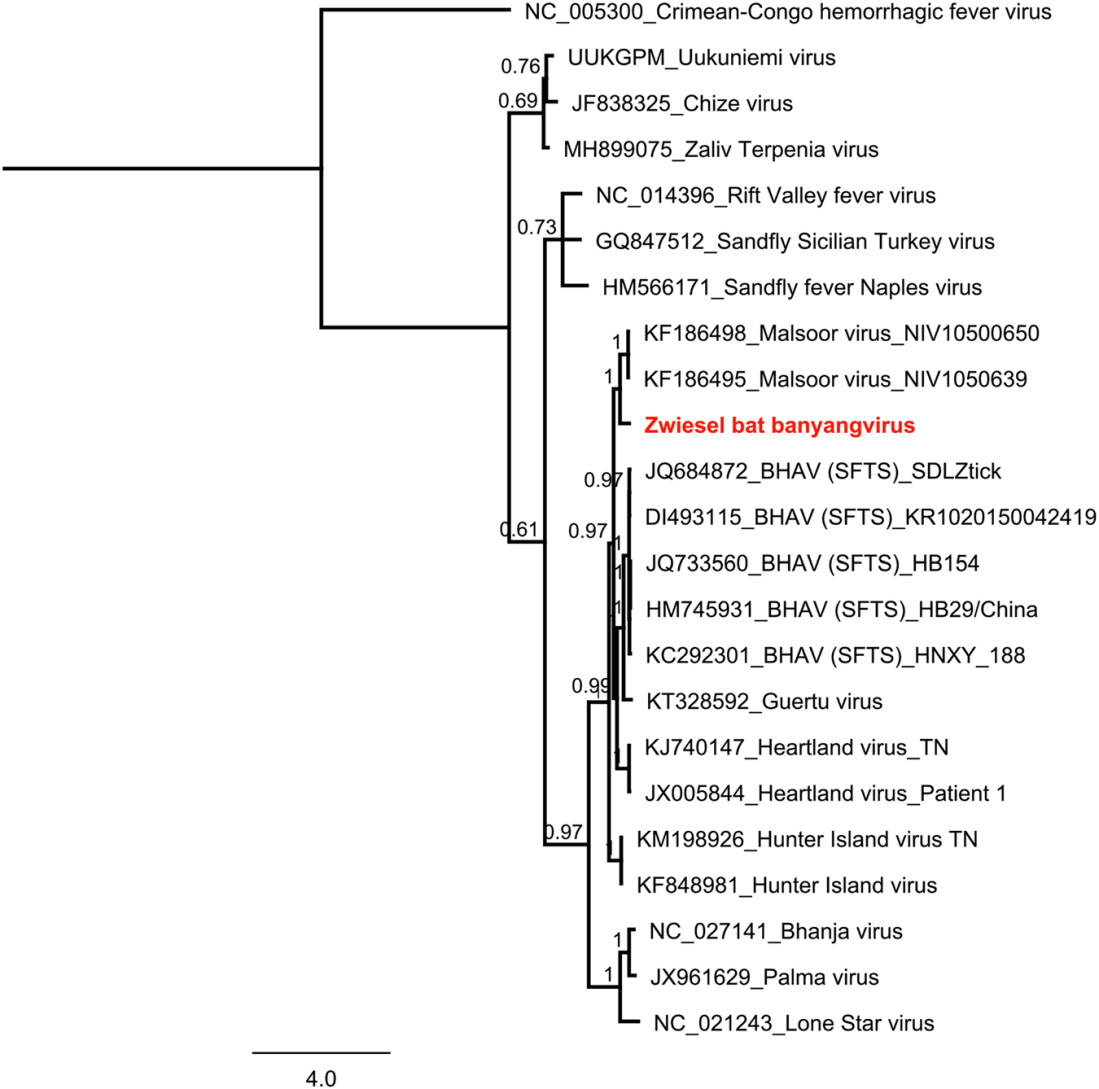
Phylogenetic analysis of the nucleotide sequence (3,094 nt) of the Zwiesel bat banyangvirus M segment. Alignments were built with ClustalW before model prediction with JModel-test. Phylogenetic tree reconstruction was calculated using MrBayes with 500,000 replicates, model GTR, sampling frequency 200, burn in 10% (50,000). Posterior probabilities are depicted at the branches.

**Figure 3:**
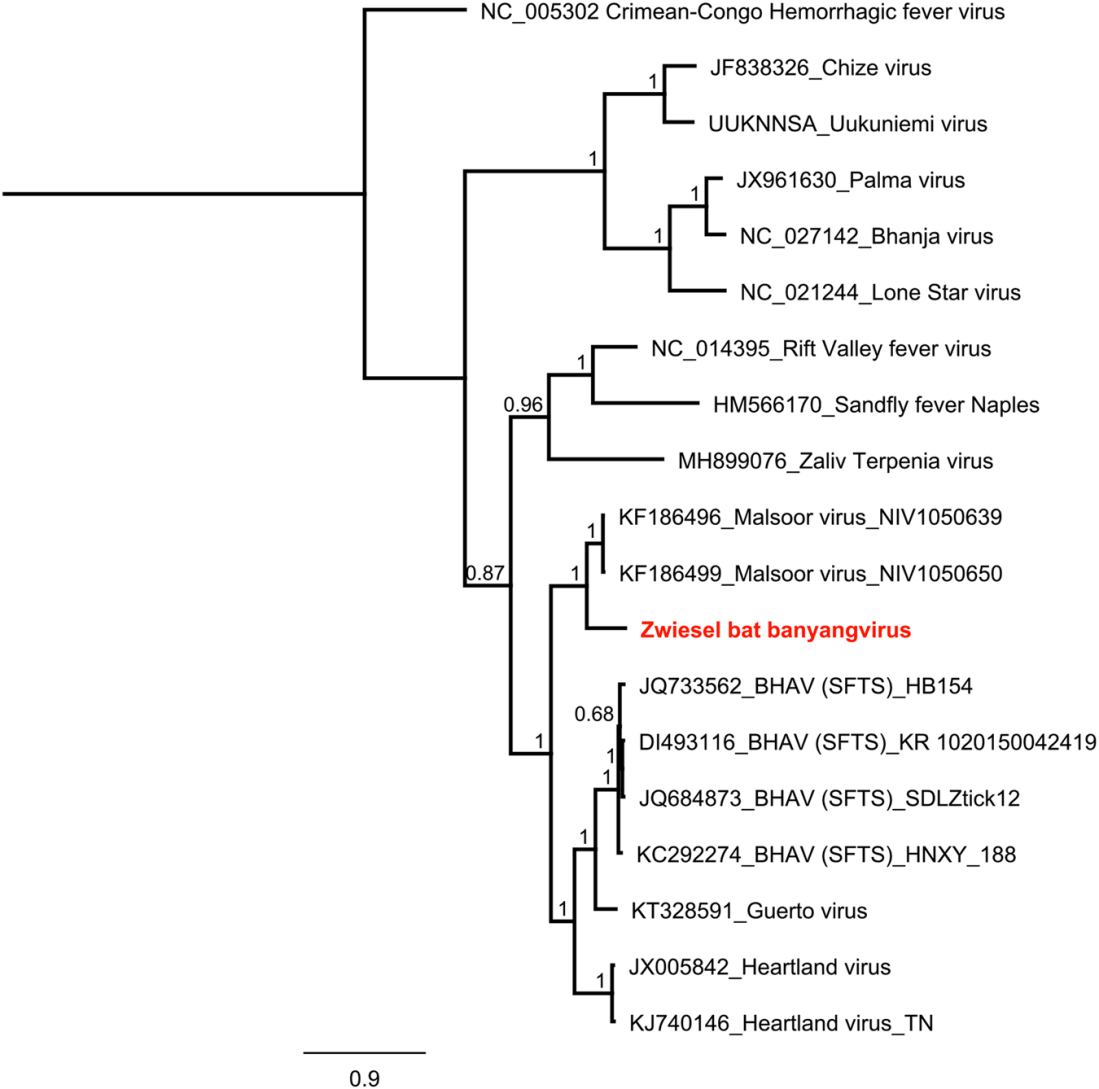
Phylogenetic analysis of the nucleotide sequence (1,793 nt) of the Zwiesel bat banyangvirus S segment. Alignments were built with ClustalW before model prediction with JModel-test. Phylogenetic tree reconstruction was calculated using MrBayes with 500,000 replicates, model GTR, sampling frequency 200, burn in 10% (50,000). Posterior probabilities are depicted at the branches.

All work was performed at Biosafety level-2 conditions with appropriate precautions. After obtaining the result that Zwiesel virus is related to SFTS virus (rated BSL-4 in Germany), further work on the original material was restricted to BSL-3. Homogenized organs [n = 31] with potentially virus-related histopathological changes (data available on request) were pooled (Table 1), followed by purification and enrichment of viruses directly from virus-infected tissue and subsequent deep sequencing of the virome^17^. Deep sequencing was performed using the Illumina HiSeq. 1500 (Illumina Inc.) technology according to the Illumina sequencing protocol. Raw reads were trimmed for primers, adapters, low-quality bases and length (>30 bp)^18^. Sequences that mapped to RefSeq sequences of eukaryotes, fungi and bacteria using Bowtie 2 version 2.2.6 with default parameters were discarded. Remaining reads were de novo assembled using Velvet assembler 1.2.10 in default mode^19,20^. Contigs and remaining sequences were aligned to the NCBI RefSeq viral protein database using DIAMOND v0.7.1^21^. All contigs and sequences with similarity to members of the genus *Banyangvirus* were assembled into larger contigs. The complete genome sequences of the L, M and S segments were obtained by using newly designed gap-filling PCR primers (sequence information available on request). Reagent concentrations were as follows: 5 µl of cDNA, 0.4 µM of each forward and reverse primer, 0.4 mM of desoxynucleoside triphosphates (Invitrogen), 2.5 ml of 10 × Platinum^®^ Taq buffer, 4 mM of MgCl_2_ and 1.25 U Platinum^®^ Taq polymerase (Invitrogen) with water added to a final volume of 25 µl. Cycling conditions were 95 °C for 10 min, 45 amplification cycles at 94 °C for 45 s, 55 °C for 45 s, 72 °C for 45 s and a final extension for 10 min at 72 °C. Open reading frames of the viral genome were predicted with Geneious 7.1.4. (http://www.geneious.com; and ClustalW by aligning to annotated phenuivirus genomes as a ref. ^22,23^. Accession numbers of the L, M and S segments used for are indicated in the respective phylogenetic trees Figs. 1, 2 and 3). Specific qPCR primers were designed to determine the viral copy numbers within the organs tested positive and to screen pooled organ tissues of an additional 363 microchiropteran bats, moribund or found dead, from the broad study (BatBanyang F: 5′ACTTCCAAAGCCACAAAAGATGGTA′3; BatBanyang R: 5′CTGAAATCGCATTAAATGTTCCATTC′3; BatBanyang MGB: 5′FAM CCTCCTAAGAAGAAATACT MGB′3^16^. Cycler conditions for qPCR were as follows: 95 °C for 10 min, 45 amplification cycles at 95 °C for 30 s, 60 °C for 30 s and 72 °C for 30 s and final extension for 10 min at 72 °C. Reagent concentrations were as follows: 1 µl of cDNA, 0.3 µM of each forward and reverse primer, 10 nM of probe, 0.1 mM of desoxynucleoside triphosphates (Invitrogen), 2.5 ml of 10 × Platinum^®^ Taq buffer, 5 mM of MgCl_2_ and 1.25 U Platinum^®^ Taq polymerase (Invitrogen) with water added to a final volume of 25 µl.

The nucleic acid sequence (6,022 nt) of the L segment, M segment (3,094 nt) and S segment (1,793 nt) where used for phylogenetic analysis. Alignments were built with ClustalW before model prediction with JModel test^23^. The model with the highest AIC value, GTR, was chosen for further analysis. Phylogenetic tree reconstruction was performed using MrBayes with 500,000 replicates, model GTR, sampling frequency 200, burn in 10% (50,000)^24–28^. Posterior probabilities are depicted at the branches.
